# Do socio-economically disadvantaged patients prefer shared decision-making?

**DOI:** 10.4102/safp.v63i1.5293

**Published:** 2021-06-17

**Authors:** Owen O. Eales, Selma Smith

**Affiliations:** 1 Department of Family Medicine, Faculty of Health Sciences, University of Pretoria, Pretoria, South Africa

**Keywords:** family medicine, preference for shared decision-making, socio-economically disadvantaged patients, chronic disease, power imbalance, patient-centeredness, communication skills

## Abstract

**Background:**

Shared decision-making is the process where patients and clinicians work together to make healthcare choices. When given a choice, most patients want to participate in decision-making about their treatment. There is a perception amongst clinicians that socio-economically disadvantaged patients do not want to participate in shared decision-making. This study investigated if patients visiting the Family Medicine Outpatient Clinic at Kalafong Hospital in Gauteng, South Africa, would prefer shared decision-making.

**Methods:**

Cross-sectional survey was performed using the Control Preference Scale. Patients visiting the Family Medicine Outpatient Clinic at Kalafong Hospital were purposively selected (*n* = 150) between February 2016 and May 2016.

**Results:**

The patients had a median age of 52 years and 53% did not finish grade 12 at school. Their median income was R3200.00 (South African Rand [ZAR]; less than $200.00) per month. Nearly half (46%) of the patients surveyed had an active preference for shared decision-making during a consultation. No demographic or disease factors had a statistically significant association with this preference.

**Conclusion:**

The perception that socio-economically disadvantaged patients do not want to actively participate in shared decision-making is incorrect according to this study. As it is not possible to predict which patients prefer an active approach to shared decision-making, it is recommended that clinicians should enquire whether they would prefer shared decision during consultations. Clinicians should also be equipped to practice this technique and an environment needs to be created that facilitates the process.

## Introduction

Shared decision-making (SDM) is a process whereby clinicians and patients work together to make healthcare choices. This process consists of three parts: (1) options are generated by both parties, (2) preferences are discussed and then (3) decisions are made together.^1^

The SDM process, where patient and clinician make shared decisions about disease management, is in stark contrast to the paternalistic attitude of the early 20th century. The American Medical Association’s original code of ethics in 1903 stated that:

The obedience of a patient to the prescriptions of his physician should be prompt and implicit. The patient should never permit his own crude opinions as to their fitness to influence his attention to them.^2^

There is an international trend towards SDM with medical bodies like the Institute of Medicine in the United States (US) and the United Kingdom (UK) General Medical Council, advising their practitioners to use SDM.^3,4^ South African public service charter Batho Pele (People First) similarly encourages SDM through its focus on consultation and participative decision-making.^5^ The trend towards SDM started in the 1970s and accelerated with the widespread access of patients to health information on the Internet in the 1990s. Patients had access to information that was previously reserved for the medical profession and this empowered patients to participate in decision-making.^2^

It is the ethical practicing of healthcare that is the main motivation behind SDM. It is the common ground between the ethical principles of beneficence and autonomy. Autonomy, the respect for the will and independence of the patient needs to be balanced with beneficence, or doing what the clinician believe is the best for the patient.^6^

The slogan of the National Health Service in the United Kingdom, ‘no decision about me without me’, is especially important in the context of chronic disease where SDM leads to increased patient participation and self-responsibility.^7^ When patients take responsibility for their own healthcare and become partners with clinicians, it leads to improved health outcomes. The advantages of SDM also include improved patient satisfaction and economic benefits to both the health system and the patient.^8^ A study conducted amongst family physicians and their patients concluded that SDM was related to lower medical expenses and decreased requests for further testing or specialist referrals.^3^

International studies show that most patients want to participate in the decision-making process regarding their medical treatment. Although there are large variations between different regions and countries, most of the patients prefer an active role in the decision-making process.^9,10,11,12^ A systematic review of SDM research shows a change in patient preferences over time towards active participation. Studies conducted after the year 2000 show a significant increase in preference for SDM compared with older studies. This indicates an increasing desire of patients to be involved in decision-making about their healthcare.^13^

International studies disagree on the association between socio-demographic factors and preference for SDM. Some studies find a significant association with preference for SDM,^8,9,10^ whilst other studies find limited association.^4,14^ However, socio-economically vulnerable patients with low levels of education and income have impaired access to quality healthcare that includes SDM. These vulnerable patients often struggle to assert their preference for SDM, which leads to physicians misunderstanding their preference to participate in SDM. This difficulty in articulating preference for SDM can be caused by time constraints in the consultation, language barrier and power imbalance between them and clinicians. A power imbalance is present between patient and doctor when the doctor has disproportionate power in relation to the patient. This is a result of the incredible power and resources given to doctors by society and health systems.^15^

Disease type plays a significant role in preference for SDM. Patients with chronic disease and cancer prefer active participation in decision-making.^11,14,16^ Chronic diseases such as diabetes are particularly appropriate for SDM because of several treatment and lifestyle options.^17^ The discipline of Family Medicine focusses on the patient rather than on the disease. This holistic approach makes SDM an approach that fits in with the values of Family Medicine.^18^ Family Medicine departments internationally are involved with research on and implementation of SDM.^1,3,13^ Recent studies from Nigerian and South African Family Medicine departments investigated family and person-centred care. It is important to note that SDM does not stand alone, but is one of the principles of person or patient-centeredness.^19,20^

No studies on the preferences of patients for SDM have been conducted in South Africa. As there is a large variation in preference between countries and regions, one cannot assume that the South African patients’ preference will be similar to recent findings in other countries.

A perception exists amongst clinicians that low-income patients do not want to participate in the decision-making process regarding their treatment.^15,21,22^ This study amongst predominantly low-income patients visiting the Family Medicine Outpatient Clinic at the Kalafong Hospital in Tshwane, South Africa, aims to bring more clarity to the issue.

The aim of this study was to describe the preferred level of SDM of South African patients using the Control Preference Scale, and to identify the demographic factors and disease types that play a role in their preferred level of SDM.

## Research methods and design

### Study design

The study was a cross-sectional survey.

### Setting

The study area was the Family Medicine Outpatient Department at Kalafong Hospital. The disease profile of these patients is similar to the national non-communicable diseases profile with cardiovascular disease, diabetes and chronic respiratory disease being the most common diagnosis.^23^

### Study population and sampling

The study population included all the patients attending the Family Medicine Outpatient Department with a chronic complaint. Purposive sampling was employed using the booking register to identify patients meeting the inclusion criteria: older than 18 years, with at least one chronic condition and fluent in any of the following languages: English, Afrikaans, Zulu or Sepedi. Patients with an acute or emergency condition and those who did not agree to be interviewed were excluded. Between 3 and 10 patients were interviewed per day from February 2016 to May 2016.

### Sample size

The events per variable of 10:1 as descibed by Perduzzi was used to determine the sample size. Five variables were investigated in this study leading to 50 events. Two goups were compared: active versus passive in an expected ratio of 2:1. This would lead to 100:50 events, or 150 participants.^24^

### Data collection

**FIGURE 1:**
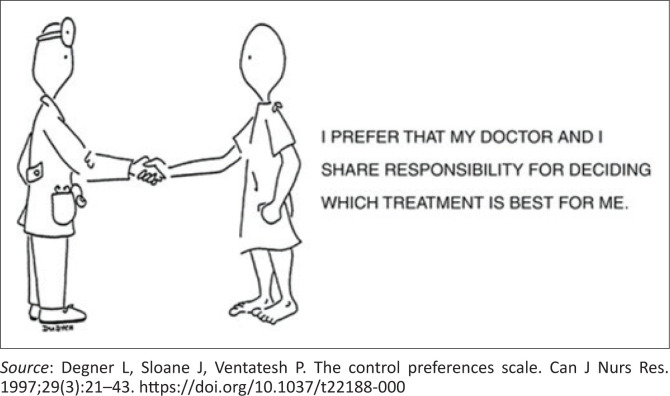
An example of a card.

The data collection tool consisted of a questionnaire design using the Control Preferences Scale in conjunction with demographic and disease accessed from the patient records. The Control Preferences Scale is a validated instrument that has been widely used and consists of cartoon pictures illustrating five different levels of SDM (Figure 1).^25^

Patient choices were documented ranging from one to five as follows: (1) I prefer to make the final selection about which treatment I will receive; (2) I prefer to make the final selection of my treatment after seriously considering my doctor’s opinion; (3) I prefer that my doctor and I share responsibility for deciding which treatment is best for me; (4) I prefer that my doctor makes the final decision about which treatment will be used, but seriously considers my opinion; (5) I prefer to leave all decisions regarding my treatment to my doctor.

The above five options can be further grouped into ‘active preference’ and ‘passive preference’ where the active preference group consists of patients choosing options 1–3. These patients prefer to be actively involved in decision-making. They either want to make the final decision or to share the decision with the doctor. Patients who are more passive in decision-making and prefer that the doctor makes the final decision about their care choose options 4 and 5.

A research assistant was trained in the use of the questionnaire and personally administered all the interviews. He was fluent in English, Afrikaans and the local languages of Zulu and Sepedi. He was observed on a weekly basis to ensure the accuracy and consistency of data collection.

### Statistical considerations

**FIGURE 2:**
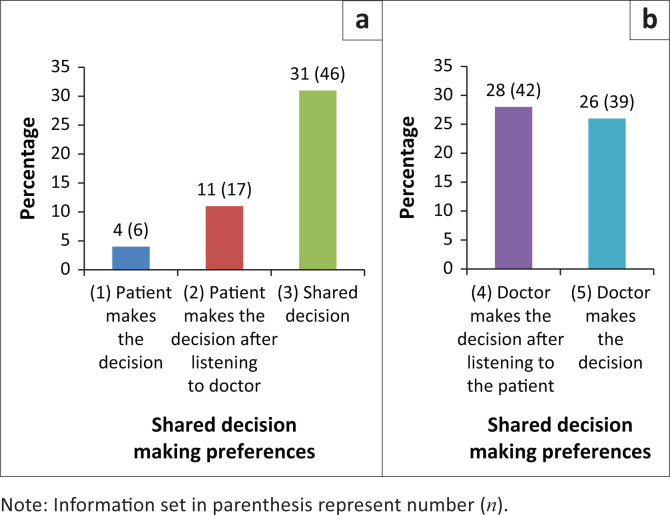
Preference for shared decision-making divided into active and passive: (a) active preference – *n* = 69 (46%) and (b) passive preference – *n* = 81 (54%).

Statistical software (StataCorp. 2015. Stata Statistical Software: Release 14. College Station, TX: StataCorp LP.) was employed for data analysis. The data that were categorical in nature and descriptive statistics frequency, percentage and 95% confidence interval (CI) were reported. Pearson’s chi-squared test was used to compare ‘passive preference’ and ‘active preference’ in contingency tables (Figure 2). Data from the contingency tables were also expressed as odds ratios (OR) along with a 95% CI. Testing was performed at the 0.05 level of significance.

### Ethical considerations

Written consent was obtained from all patients prior to interview. Permission to conduct the research was received from the Chief Executive Officer of Kalafong hospital. Ethical approval was obtained from the Faculty of Health Sciences Research Ethics Committee of the University of Pretoria (Protocol no. 503/2015).

## Results

### Demographics

In this study (*n* = 150), the number of male and female patients were more or less equal and the median age of interviewees was 52 years. Nearly half of the study population completed grade 12 or higher qualification. More than 40% of patients had an income level of less than R1500.00 (Table 1).

**TABLE 1:** Demographic and disease profile.

Variable	*n*	%
**Sex (*n* = 150)**		
Male	74	49.0
Female	76	51.0
**Age (years) (*n* = 150)**		
Average age	51	-
Median age	52	-
60 years and older	52	34.7
**Education level (*n* = 141)**		
Less than Grade 8	38	27.0
Between Grade 8 and Grade 12	36	25.5
Completed Grade 12	52	36.9
Higher education	15	10.6
**Income (*n* = 121)**		
< R1500.00	50	41.3
R1500.00 – R10 000.00	47	38.8
> R10 000.00	24	19.8
**Disease profile (*n* = 150)**		
More than one disease	84	56.0
Diabetes	61	40.6
Hypertension	60	40.0
Arthritis	33	22.0
Epilepsy	29	19.3
Diabetics with hypertension	24	16.0
Other	5	3.3
Psychiatric	2	1.3

### Disease profile

Diabetes (40.6%), hypertension (40.0%) and arthritis (22.0%) were identified as the most common chronic diseases. More than half (56.0%) of the patients had more than one chronic disease.

#### Preferred level of shared decision-making

The results for the different options are depicted by Figure 2. Most patients preferred the balanced SDM option 3 (31%) or the passive preference options 4 or 5.

For further statistical analysis and for comparison to international literature, the five options were collapsed into two groups, namely those with active preference and those with passive preference. A total of 46% of patients preferred to be actively involved in decision-making. They either wanted to make the final decision or to share the decision with the doctor. Fifty-four percent of patients were more passive in decision-making and preferred the doctor to make the final decision about their care.

#### Demographic and disease factors that influence the preferred level of shared decision-making

In Table 2, it is clear that no demographic factors showed any statistically significant relationship (*p* < 0.05) with the preference for SDM; however, age and income level were found to have a marginally significant association (0.1 > *p* > 0.05).

**TABLE 2:** Association between demographic or disease parameters with ‘passive’ preferred level of shared decision-making.

Exposure	Passive (*n*)	%	*p*-Value (chi-squared)	Odds ratio	95% CI
**Sex (*n* = 150)**					
Male (*n* = 74)	33	55.4	-	1.00	-
Female (*n* = 76)	36	52.6	0.73	0.89	0.45–1.7
**Age (*n* = 150)**					
≤ 45 (*n* = 48)	22	45.8	0.08 (Marginal)	1.00	-
> 45–60 (*n* = 51)	25	49.0		1.14	0.51–2.51
> 60 (*n* = 61)	34	66.8		2.36	1.02–5.45
**Education (*n* = 141)**					
< Gr 12 (*n* = 74)	44	59.5	0.06 (Marginal)	1.00	-
≥ Gr 12 (*n* = 74)	29	43.0		0.52	0.26–1.03
**Income (monthly) (*n* = 121)**					
< R1500.00 (*n* = 50)	29	58.0	-	1.00	-
R1500.00 – R10 000.00 (*n* = 47)	27	57.5	-	0.98	0.43–2.98
> R10 000.00 (*n* = 24)	10	41.7	0.37	0.51	0.19–1.41
**Disease (*n* = 150)**					
Non-diabetic (*n* = 87)	43	49.4	-	1.00	-
Diabetic (*n* = 61)	36	59.0	0.31	1.47	0.76–2.87

CI, confidence interval.

There was no statistical association between disease type and preferred level of SDM in the Kalafong study. Diabetics were compared with patients with other chronic conditions non-diabetics and no statistical significant difference was observed.

## Discussion

### Key findings

It is noteworthy that nearly half of the chronic patients sampled (46%) preferred to actively participate in the decision-making process. No demographic of disease factors was significantly associated with preference for SDM, although younger age and higher education levels were marginally associated with active preference for SDM.

### Discussion of key findings

Although the preference for active decision-making in the Kalafong study is less than other international studies,^4,10,12^ it is important to note that it is the first study on the preference for SDM in South Africa, and that the participants had a lower education level compared to the other studies. Previous research had shown that less educated patients are more passive in their preference for SDM.^9^

The fact that nearly half of all patients wanted to play an active role in SDM is in contrast to perceptions that socio-economically disadvantaged patients do not want to participate in SDM.^21,22^

This perception is likely caused by patients not clearly articulating their preference for SDM. Factors such as the language barrier, lack of time per consultation, and a power imbalance between patients and clinician may impede the patients’ ability to clearly communicate their preferences, and will be discussed in more detail.^26^

The language barriers that exist between clinicians and patients in the South African public health sector could contribute to this perception. The accepted language used in the healthcare setting is English, which is often not the mother tongue for either the patient or the clinician. This is a significant barrier to the expression of the need for SDM by the patient and the ability of the clinician to understand this need.^27^

The lack of time available during routine consultation can also lead to breakdown of communication and an inability of the patient to express their treatment preferences.^27^ The workload of clinicians in the public sector is often very high with some clinicians seeing up to 60 patients per day.^28^ Under these circumstances, a consultation can only take a few minutes, which does not leave room for patients to express their preferences.

Another reason for patients not to express their need for participation in SDM is the power imbalance between clinicians and patients. A power imbalance exists between two parties when one party has disproportionate power in relation to the other. Clinicians have been given extraordinary resources, status and power by society, in contrast to patients who are fearful and in desperate need for help.^26^ This imbalance may contribute to an inability of the patient to communicate their preference for SDM. Even an empathetic clinician could interpret this silence as a preference not to participate in the decision-making process.^15^ In the South African public service, there is a significant power imbalance between patients and clinicians. Patients who attend public health facilities are mostly uninsured and often unemployed or pensioners. The clinicians, on the other hand, have job security, a good income and mostly have a privileged background.^29^ This divide between the socio-economic circumstances of clinicians and patients could increase the power imbalance and worsen the patients’ ability to express their preferences.

The lack of a statistically significant association between socio-demographic factors and preferred level of SDM in the Kalafong study is of interest. Younger age and higher education levels were found to have only marginally significant association for active decision-making. These results are similar to international studies that find only a limited association between these factors and preference for SDM.^4,14^

It was expected that the diabetic group would have a higher preference for active SDM because of the high patient involvement and extensive patient education needed to manage diabetes, but this was not found.

A reason for the lack of association between socio-demographic factors and disease type with the preference for SDM in the Kalafong group could be because of the relative uniformity of the group. It was an older group, less than half of them had completed secondary school and their average income was less than $200.00 (R3100.00) per month. The older age, low-income and education levels could affect their preference for SDM and neutralise other factors such as sex and disease type.

## Limitations

In this study, certain personalities and disease types could be misrepresented because of the time of day and day of the week the interviews took place. To address these, interviews were performed on all the days of the week and at different times in an attempt to minimise selection bias. Random or systematic sampling minimise bias and would have been more appropriate than purposive sampling for this type of study. The preferred level of SDM amongst private practice patients in South Africa and the comparison with the public sector were not studied. It could be that private patients are more autonomous and consumerist as people pay for their medical services. The study focuses on chronic, non-communicable diseases and excludes the huge percentage of human immunodeficiency virus–positive patients who are on chronic medication. The findings of this study can therefore not be generalised to patients with acute or communicable diseases. The cartoon cards portray a hospital-based scenario and could be misunderstood in a clinic setting.

## Recommendations

It is difficult to predict the preference of patients for SDM and assumptions on the part of clinicians are often wrong. It is therefore imperative that the clinician asks patients about their preference for SDM.

Significant system changes also need to be implemented in the public health sector to enable clinicians and patients to share in the decision-making process. Clinicians are often overwhelmed by patient numbers and feel like they must just ‘push the queue’ of finish the patients waiting to see them.^30^ More time needs to be structured between clinician and patients to facilitate meaningful communication. An efficient booking system for patients could contribute to structuring the time per patient for clinicians.

The application of SDM especially amongst patients with non-communicable disease could empower patients and decrease the burden on the public health system.

Because of the involvement of family medicine with primary healthcare and its focus on holistic patient care, the discipline is uniquely positioned to champion SDM and to teach it to both undergraduate and postgraduate students.

## Conclusion

A significant percentage of chronic patients at the Kalafong Family Medicine Outpatient Department prefer to participate in decision-making regarding their treatment. It is difficult to predict which patients want to participate; therefore, it is recommended that the patients are asked for their preference and create an environment where SDM can take place. Clinicians are encouraged to remember the quote ‘no decision about me without me’.
